# Leveraging National Health Survey Data for Life-Course Analysis: Socioeconomic Positioning and Adult Obesity in South Korea Using KNHANES

**DOI:** 10.23889/ijpds.v11i3.3430

**Published:** 2026-09-29

**Authors:** Zhansulu Issayeva, Ki-Soo Eun

**Affiliations:** 1 Graduate School of International Studies, Seoul National University, Seoul, Republic of Korea

**Keywords:** life-course approach, socioeconomic status, obesity, KNHANES, linked data, health inequalities

## Abstract

This study examines how nationally representative health survey data can be used to operationalise a life-course approach to health inequalities by examining socioeconomic inequalities and adult obesity in South Korea. Drawing on the life-course framework and using data from the ninth wave of the Korea National Health and Nutrition Examination Survey (KNHANES, 2022–2024), the study explores how multiple indicators of socioeconomic status (SES) are associated with obesity among adults aged 19 years and older (n=12,075). South Korea provides a valuable context for life-course research as a high-income country that has undergone rapid socioeconomic and demographic transformation within a single generation. KNHANES is one of the most comprehensive nationally representative health surveys in Asia and includes detailed information on education, income, employment, and demographic characteristics. Although cross-sectional, the dataset enables the examination of socioeconomic positioning and cumulative socioeconomic disadvantage using proxy indicators measured in adulthood. Obesity, defined using the Korean standard body mass index threshold (BMI ≥25 kg/m^2^), is analysed as a key outcome associated with long-term socioeconomic conditions. The findings reveal clear socioeconomic patterning in adult obesity. Lower educational attainment emerged as the most consistent predictor of obesity, while income and employment status showed more heterogeneous associations after adjustment. These findings are consistent with a life-course perspective in which cumulative socioeconomic conditions and structural constraints may shape obesity risk among adults. By demonstrating how nationally representative survey data can support life-course analysis in the absence of fully linked longitudinal systems, this study highlights the potential of existing population health datasets to inform future data linkage initiatives and comparative research on health inequalities. The findings contribute to ongoing efforts to leverage routinely collected data to support life-course approaches to improving population health and wellbeing.

## Introduction

In recent years, there has been growing recognition of the importance of adopting a life-course approach to understanding health and wellbeing. The life-course perspective emphasises that health outcomes are shaped by cumulative exposures and experiences across different stages of life rather than by isolated events or behaviours [1, 2]. Health outcomes are therefore understood as dynamic processes influenced by social, economic, and environmental determinants over time, with increasing policy attention given to operationalising life-course approaches to support healthy ageing and population wellbeing [3, 4]. A central component of advancing life-course research is the availability and effective use of linked data. Linked data systems enable integration of information from multiple sources, including health records, administrative data, and social service databases, allowing researchers to examine how exposures at one stage of life influence outcomes at later stages and to identify patterns of inequality across populations [5, 6]. However, in many countries, although linked administrative data infrastructures have expanded globally, access and integration remain uneven across countries and institutional settings [7]. In this context, repeated cross-sectional nationally representative health surveys provide an important complementary resource for research adopting a life-course perspective, particularly in settings where fully integrated longitudinal or linked administrative data systems remain limited. Although cross-sectional in design, these surveys contain detailed information on socioeconomic conditions, health behaviours, and demographic characteristics that can support analyses within life-course framework by capturing multiple dimensions of socioeconomic and demographic positioning. Analysed through a life-course lens, such datasets offer valuable insights into how socioeconomic conditions and structural determinants may interact to shape health outcomes over time, even when direct longitudinal processes cannot be directly observed [8].

South Korea offers a particularly relevant context for examining the potential of national health survey data in life-course research. Over recent decades, the country has undergone rapid socioeconomic transformation accompanied by significant shifts in population health patterns. While life expectancy and healthcare access have improved substantially, South Korea faces growing challenges related to non-communicable diseases, including obesity [9, 10]. Obesity prevalence has increased steadily in recent years, reflecting broader changes in dietary patterns, physical activity, and socioeconomic conditions [11]. At the same time, health inequalities associated with education, income, and employment have become more pronounced, underscoring the importance of a life-course perspective in public health research and policy.

The Korea National Health and Nutrition Examination Survey (KNHANES) is one of the most comprehensive nationally representative health surveys in Asia. Conducted by the Korea Disease Control and Prevention Agency, KNHANES collects detailed information on health status, behaviours, nutrition, and socioeconomic characteristics through interviews, examinations, and laboratory assessments [12]. The ninth wave of KNHANES (2022–2024) represents the most recent available dataset and provides an important opportunity to examine contemporary health inequalities in South Korea within a life-course analytical framework. Although KNHANES is not a longitudinal panel dataset, it includes a wide range of socioeconomic and demographic variables that can be used to construct indicators of life-course socioeconomic status. Education, household income, employment status, and marital status reflect different dimensions of socioeconomic positioning associated with health outcomes [13]. When analysed together, these variables provide insight into how socioeconomic disadvantage may shape obesity risk and broader health outcomes.

This study therefore leverages data from the ninth wave of KNHANES to examine socioeconomic positioning and adult obesity within a life-course framework. Specifically, it assesses the association between socioeconomic indicators and obesity among adults aged 19 years and older in South Korea, demonstrates how nationally representative cross-sectional survey data can be used within a life-course framework in the absence of fully linked longitudinal datasets, and discusses how the findings may inform future linkage of national health surveys with administrative or sectoral datasets to support life-course research and policy development. By situating the analysis within broader life-course health research and ongoing efforts to improve data integration across sectors, this study contributes to discussions on how routinely collected population data can be leveraged to understand patterns of health inequalities and inform multisectoral strategies for improving health and wellbeing across the life-course.

## Literature Review and Conceptual Framework

### The Life-Course Approach to Health and Wellbeing

The life-course approach to health has emerged as a central paradigm in contemporary public health and population health research. Rooted in interdisciplinary traditions spanning epidemiology, sociology, demography, and social policy, the life-course perspective emphasises that health outcomes are shaped by cumulative exposures and experiences across different stages of life rather than by isolated risk factors occurring at a single point in time [14, 15]. This perspective aligns closely with evolving international life-course frameworks that highlight the importance of promoting functional ability, resilience, and wellbeing across all ages, from early development to healthy ageing [3].

Within this framework, health outcomes are understood as being shaped by biological, behavioural, and social determinants operating across the lifespan. Early-life conditions, including family socioeconomic status, education, and nutrition, play a foundational role in shaping later health outcomes by influencing physical development, cognitive capacities, and health behaviours [16]. These early influences interact with subsequent exposures in adolescence and adulthood, including educational attainment, employment opportunities, and social environments, contributing to cumulative patterns of advantage and disadvantage in health. Three primary models are commonly used to conceptualise life-course influences on health: the critical period model, the accumulation of risk model, and the pathway model [14]. These models are not mutually exclusive and often operate simultaneously, underscoring the complexity of life-course health dynamics.

In the context of non-communicable diseases such as obesity, the life-course approach is particularly relevant. Obesity is influenced by long-term patterns of diet, physical activity, and socioeconomic conditions that evolve over time. Understanding how these factors interact across different life stages is essential for designing effective prevention strategies that extend beyond individual behaviour change to address structural determinants of health [17].

### Socioeconomic Status and Health Inequalities Across the Life-Course

Socioeconomic status (SES) is widely recognised as a fundamental determinant of health. It encompasses multiple dimensions, including education, income, occupation, and wealth, which shape access to resources, opportunities, and social environments that influence health outcomes [6]. Extensive evidence documents strong and persistent associations between SES and morbidity, mortality, and self-rated health [18]. From a life-course perspective, socioeconomic inequalities in health arise through cumulative and pathway mechanisms. Individuals from disadvantaged backgrounds are more likely to experience adverse early-life conditions and limited opportunities for upward mobility, with effects that persist into adulthood and shape health behaviours and access to care [19].

Education plays a central role in shaping health outcomes across the life-course. Higher educational attainment is associated with greater health literacy, improved employment prospects, and higher income, all of which contribute to better health outcomes [20]. In contrast, lower education is often linked to precarious employment and increased exposure to health risks. Household income reflects the material resources available for maintaining healthy living conditions and accessing healthcare [21]. Employment status and working conditions also influence health across the life-course. Stable employment provides financial security and social integration, whereas unemployment or precarious employment is associated with financial stress and adverse health outcomes [22]. Marital status and household composition further shape socioeconomic and psychosocial environments, influencing health behaviours and social support networks [23]. Importantly, these socioeconomic dimensions interact over time, contributing to cumulative patterns of advantage and disadvantage that shape health outcomes throughout adult life. Persistent socioeconomic disadvantage across life stages increases the risk of adverse outcomes, including obesity and related chronic conditions.

### Obesity as a Life-Course Health Outcome

Obesity has emerged as one of the most pressing public health challenges globally. It is associated with increased risk of cardiovascular disease, diabetes, certain cancers, and reduced quality of life [24]. While obesity has traditionally been more prevalent in high-income Western countries, its prevalence has risen rapidly in many Asian contexts, including South Korea, reflecting broader socioeconomic and lifestyle changes [25].

The determinants of obesity are complex and multifactorial, encompassing genetic, behavioural, environmental, and socioeconomic factors. From a life-course perspective, obesity is shaped by long-term patterns of energy balance influenced by diet, physical activity, and metabolic processes [26]. Early-life nutrition, childhood socioeconomic conditions, and educational attainment play important roles in shaping lifelong health behaviours and metabolic health. Socioeconomic inequalities in obesity have been widely documented, although their patterns vary across countries and population groups. In rapidly developing economies, the relationship between SES and obesity may shift as countries undergo economic and nutritional transitions [27].

South Korea represents an important case in this regard. Rapid economic development and urbanisation have transformed dietary patterns, physical activity levels, and occupational structures, contributing to changes in obesity prevalence. Previous studies in South Korea have documented significant socioeconomic inequalities in obesity, although findings have varied depending on the socioeconomic indicator examined [21]. Educational attainment has consistently emerged as one of the strongest predictors of obesity, particularly among women, while associations with household income and occupation have been less consistent [28]. Research using earlier waves of KNHANES has shown that lower educational attainment is associated with a higher prevalence of obesity and related metabolic risk factors, suggesting that socioeconomic influences on obesity extend beyond current material resources alone [29]. These findings highlight the importance of examining multiple dimensions of socioeconomic positioning simultaneously rather than relying on a single SES indicator. Understanding how socioeconomic factors across the life-course influence obesity in the Korean context is therefore essential for informing targeted public health interventions.

### Leveraging National Health Survey Data for Life-Course Research

The growing emphasis on life-course approaches to health underscores the need for data systems that capture exposures and outcomes across different stages of life. Linked administrative datasets integrating information from health, education, employment, and social services provide powerful tools for examining life-course research by enabling researchers to track individuals across time and sectors. Despite their analytical value, such comprehensive linked data infrastructures remain unevenly developed across countries. However, linkage across institutional datasets in South Korea remains methodologically and administratively challenging due to fragmented governance structures, differing access procedures across agencies, privacy regulations, and concerns regarding secure data sharing and long-term data stewardship. In many settings, including South Korea, administrative data are collected by separate agencies and are not routinely linked for research purposes. Privacy regulations, institutional barriers, and technical challenges further limit access to integrated datasets, meaning researchers often rely on cross-sectional survey data to examine health inequalities and their determinants [30]. In the Korean context, future linkage between national health survey data and administrative datasets such as National Health Insurance Service records, employment histories, educational records, mortality data, and neighbourhood-level contextual data could substantially strengthen the ability to examine long-term socioeconomic influences on health. National health surveys such as KNHANES provide valuable resources for life-course research, even in the absence of longitudinal linkage. These surveys typically include detailed information on socioeconomic characteristics, health behaviours, and health outcomes that can be analysed within a life-course framework. While they do not track individuals over time, they capture snapshots of different age cohorts and socioeconomic groups, allowing for the examination of cumulative patterns of disadvantage and health risk [12]. Moreover, national surveys can serve as foundational components of future linked data systems. By identifying key variables and data gaps, survey-based research can inform efforts to integrate data across sectors and improve the availability of longitudinal information for life-course analysis. In this sense, leveraging existing survey data is not only a pragmatic approach to studying health inequalities but also a step toward developing more comprehensive linked data infrastructures.

### Study Rationale and Contribution

Despite the growing recognition of the importance of life-course approaches to health, relatively few studies have examined socioeconomic positioning and obesity within a life-course framework using nationally representative data from South Korea. While previous Korean studies have examined individual SES indicators, fewer studies have examined multiple dimensions of socioeconomic positioning within an explicit life-course framework. Existing research has often focused on single indicators of SES or specific age groups, limiting understanding of how multiple socioeconomic factors interact to shape health outcomes. Furthermore, there is limited discussion of how national health survey data can be leveraged for life-course research and integrated into broader linked data systems [18].

This study addresses these gaps by using data from KNHANES 9^th^ wave (2022–2024) to examine the relationship between socioeconomic indicators and adult obesity within a life-course framework. By analysing multiple dimensions of SES simultaneously, the study provides a more comprehensive understanding of how socioeconomic dimensions are associated with obesity risk. In addition, it situates the analysis within the broader context of linked data and life-course research, highlighting opportunities and challenges for enhancing data integration and policy relevance.

Through this approach, the paper contributes to ongoing efforts to operationalise life-course frameworks in public health research and to leverage routinely collected data for improving health and wellbeing across populations. It also offers insights relevant to other countries seeking to strengthen data systems and address health inequalities through a life-course perspective.

## Methods

### Data source: Korea national health and nutrition examination survey (KNHANES 9^th^ wave)

This study uses data from the ninth wave of the Korea National Health and Nutrition Examination Survey (KNHANES 9th wave), covering the period 2022–2024. KNHANES is a nationally representative, cross-sectional survey conducted by the Korea Disease Control and Prevention Agency (KDCA) to monitor the health and nutritional status of the Korean population [31]. Since its inception in 1998, KNHANES has served as a key source of population health data for research and policy development in South Korea. KNHANES employs a stratified, multistage probability sampling design to ensure representativeness of the non-institutionalised civilian population [31]. Sampling units are selected based on geographic region, sex, and age distribution using census data. The survey consists of three primary components: a health interview, a health examination, and a nutrition survey. Together, these components provide comprehensive information on demographic characteristics, socioeconomic status, health behaviours, clinical indicators, and nutritional intake.

The ninth wave of KNHANES represents the most recent fully available dataset at the time of analysis [32]. As with previous waves, data collection includes measured anthropometric indicators such as height and weight, enabling accurate calculation of body mass index (BMI). The survey also collects detailed information on education, household income, employment, marital status, and residential characteristics, which are essential for examining socioeconomic determinants of health within a life-course framework.

### Study Population

The analytical sample for this study consists of adults aged 19 years and older who participated in KNHANES 9^th^ wave (2022–2024). Individuals with missing data on key variables, including BMI or socioeconomic indicators, were excluded from the final analysis. Both men and women were included to capture gender differences in socioeconomic patterns and obesity prevalence. The use of a nationally representative adult sample allows for examination of socioeconomic gradients in obesity across the broader Korean population. Sampling weights provided by KNHANES were applied in all analyses to account for the complex survey design and to produce estimates representative of the national population.

### Outcome Variable: Obesity

The primary outcome variable in this study is obesity status. Obesity was defined using body mass index (BMI), calculated as weight in kilograms divided by height in metres squared (kg/m^2^). Height and weight measurements in KNHANES are obtained through standardised health examinations conducted by trained personnel, ensuring high reliability and validity. Consistent with Korean and international public health guidelines, obesity was defined as BMI ≥25 kg/m^2^ for Asian populations, reflecting evidence that obesity-related health risks occur at lower BMI thresholds than in Western populations [33]. Individuals with BMI below 25 kg/m^2^ were classified as non-obese for the purposes of analysis. A binary variable indicating obesity status was constructed to facilitate logistic regression modelling.

### Socioeconomic Indicators and Life-Course SES Construction

To examine socioeconomic inequalities from a life-course perspective, this study incorporates multiple indicators of socioeconomic status (SES), including educational attainment, household income, employment status, marital status, and region of residence. Together, these variables capture key dimensions of current and cumulative socioeconomic positioning that influence health over time. The construction and life-course interpretation of these indicators are summarised in Table 1.

**Table 1:** Construction of Life–Course Socioeconomic Status Indicators Using KNHANES 9^th^ Wave (2022–2024)

**Dimension of SES**	**Variable (KNHANES)**	**Categories Used in Analysis**	**Life-Course Interpretation**
Educational attainment	Highest level of education completed	Elementary or less; Middle school; High school; College or higher	Proxy for early-life and cumulative socioeconomic conditions shaping adult opportunities and health behaviours
Household income	Equivalised household income quartiles	Lowest; Lower-middle; Upper-middle; Highest	Indicator of current material socioeconomic resources influencing living conditions, diet, and health access
Employment status	Current employment status	Employed; Not employed	Reflects adult socioeconomic stability, income security, and social integration
Marital status	Marital status	Married; Never married; Previously married (divorced/widowed/separated)	Captures social support structures and household-level socioeconomic context
Region of residence	Administrative region classification	Urban; Rural	Contextual socioeconomic and environmental exposure influencing health behaviours and access to services

Educational attainment and household income were included as core indicators of socioeconomic positioning. Educational attainment was categorised into four groups (elementary school or less, middle school, high school, and college or higher) and serves as a proxy for earlier-life and cumulative socioeconomic conditions that may influence health behaviours and opportunities throughout adulthood. Household income was measured using equivalised household income quartiles (lowest, lower-middle, upper-middle, and highest), reflecting current material resources and living conditions.

Employment status, marital status, and region of residence were included to capture additional dimensions of adult socioeconomic and social context. Employment status was classified as employed or not employed and reflects economic stability and social integration. Marital status was categorised as married, never married, or previously married (divorced, separated, or widowed), capturing differences in household structure and social support. Region of residence was classified as urban or rural and serves as a contextual indicator of environmental conditions, healthcare access, and health-related resources.

### Analytical Strategy

#### Statistical Analysis

Descriptive statistics were first calculated to summarise the distribution of demographic and socioeconomic characteristics within the study population. Obesity prevalence was estimated across categories of education, income, employment status, marital status, and region of residence. Differences in obesity prevalence across socioeconomic groups were examined to identify patterns of inequality. To assess the association between socioeconomic indicators and obesity, logistic regression models were employed. The dependent variable was obesity status (BMI ≥25 kg/m^2^), coded as a binary outcome. Independent variables included education, household income, employment status, marital status, and region of residence, with age and sex included as control variables. These variables were selected based on the study’s life-course framework, previous literature on socioeconomic inequalities in obesity, and the availability of relevant measures within KNHANES. A series of stepwise models were estimated to examine the independent and combined effects of socioeconomic indicators. Model 1 included demographic variables (age and sex). Model 2 added educational attainment and household income. Model 3 further incorporated occupational categories, marital status, and region of residence. This approach enabled assessment of how different dimensions of SES contribute to obesity risk. All analyses were conducted using survey weights and appropriate procedures to account for the complex sampling design of KNHANES. Analyses were performed using Stata/IC 16.1 (StataCorp LLC, College Station, TX, USA). Survey design features, including sampling weights, stratification, and primary sampling units, were incorporated using the svy procedures. Prior to model estimation, the relationships among explanatory variables were assessed to minimise potential multicollinearity concerns, and no evidence of problematic overlap among the included socioeconomic indicators was identified. Odds ratios (ORs) and 95% confidence intervals (CIs) were reported. The model-building strategy was theory-driven and designed to reflect different dimensions of socioeconomic positioning. Model 1 included age and sex as basic demographic covariates. Model 2 added educational attainment and household income, representing relatively stable socioeconomic positioning and current material resources. Model 3 further incorporated occupational category, marital status, and region of residence to account for adult social, labour market, and contextual conditions. This sequential approach allowed assessment of whether associations between education and obesity persisted after adjustment for current socioeconomic and demographic circumstances.

#### Leveraging Cross-Sectional Data for Life-Course Analysis

Although KNHANES is a cross-sectional survey, it contains rich socioeconomic and demographic information that can support analyses within a life-course framework. Rather than directly observing longitudinal socioeconomic processes, this study examines multiple socioeconomic indicators, including education, income, and occupation, as complementary proxy measures of socioeconomic positioning [34]. Educational attainment is interpreted as a relatively stable marker reflecting earlier-life socioeconomic opportunities and social positioning, while income and employment status reflect current adult socioeconomic circumstances. This approach aligns with life-course epidemiological perspectives emphasising cumulative disadvantage and pathway mechanisms, while acknowledging the inferential limitations of cross-sectional data. Accordingly, the analysis does not directly observe longitudinal socioeconomic change, but instead examines patterns consistent with cumulative socioeconomic influences on obesity risk. The analytical approach used in this study may also help identify priority domains for future linked data research, including longitudinal educational, employment, healthcare utilisation, and neighbourhood-level records capable of capturing socioeconomic exposures across different stages of life.

## Results

### Sample Characteristics

The analytical sample consisted of adults aged 19 years and older drawn from the ninth wave of the Korea National Health and Nutrition Examination Survey (KNHANES 2022–2024). After excluding respondents with missing data on body mass index or key socioeconomic variables, the final weighted sample was nationally representative of the non-institutionalised adult population of South Korea [35]. After appending the 2022–2024 datasets and applying inclusion/exclusion criteria (age ≥19; non-missing BMI, SES variables; income quartiles 1–4), the final analytic sample included n = 12,075 respondents (unweighted). Using KNHANES sampling weights, the sample represents the national adult population of South Korea. The weighted obesity prevalence (BMI ≥25 kg/m^2^) was 38.0%.

Table 2 presents the descriptive characteristics of the study population. The sample included both men and women across diverse age groups and socioeconomic backgrounds. A substantial proportion of respondents had completed tertiary education, reflecting South Korea’s high educational attainment levels. However, notable variation remained across age cohorts, with lower educational attainment more common among older adults. Household income was distributed across quartiles, with representation across all socioeconomic strata. Most working-age adults were employed, although employment rates varied by gender and age. The majority of respondents were married, while a smaller proportion were never married or previously married. Consistent with national demographic patterns, most respondents resided in urban areas. Overall obesity prevalence (BMI ≥25 kg/m^2^) indicated that a considerable proportion of Korean adults were classified as obese, highlighting obesity as a significant public health concern.

**Table 2:** Weighted Sample Characteristics of Adults Aged ≥19 years, KNHANES 9^th^ Wave (2022–2024)

**Variable**	**Category**	**n**	**Weighted %**
Sex	Men	5,202	49.9
	Women	6,873	50.1
Age group	19–29	1,373	16.1
	30–44	2,474	25.1
	45–64	4,625	37.2
	≥65	3,603	21.6
Education	Elementary or less	2,153	12.1
	Middle school	1,238	9.5
	High school	4,112	35.2
	College+	4,486	43.2
Household income (incm5)	Lowest	2,944	24.0
	Lower-middle	3,046	24.9
	Upper-middle	3,048	25.0
	Highest	3,037	26.1
Employment (occp)	Employed (1–6)	7,429	65.5
	Not employed (7)	4,646	34.5
Marital status (marri_1)	Married	9,806	74.3
	Not married	2,269	25.7
Region (town_t)	Urban	9,322	82.7
	Rural	2,753	17.3
Obesity	Obese (BMI ≥25)	4,496	38.0
	Not obese	7,579	62.0

Unweighted n shown; weighted % shown.

### Obesity Prevalence by Socioeconomic Status

Obesity prevalence varied significantly across socioeconomic groups (Table 3). Clear gradients were observed by education and household income. Individuals with lower educational attainment exhibited higher obesity prevalence (41.2%) compared with those holding tertiary education (37.4%). Differences by income quartile were modest in unadjusted comparisons. Employed adults had higher obesity prevalence (40.1%) than those coded as not employed (34.0%).

Married individuals, particularly men, showed higher prevalence compared with never married individuals. Rural residents exhibited slightly higher obesity prevalence than urban residents, suggesting potential contextual influences related to environment and access to health-promoting resources. These descriptive findings indicate the presence of socioeconomic inequalities in obesity within the Korean adult population.

**Table 3:** Obesity Prevalence (BMI ≥25) by Socioeconomic Characteristics

**Variable**	**Category**	**n**	**Obesity %**	**95% CI**
Education	Elementary or less	2,153	41.2	38.8–43.6
	Middle school	1,238	39.3	36.2–42.5
	High school	4,112	37.5	35.7–39.3
	College+	4,486	37.4	35.8–39.0
Income quartile	Lowest	2,944	38.2	36.1–40.2
	Lower-middle	3,046	38.8	36.9–40.6
	Upper-middle	3,048	37.7	35.8–39.6
	Highest	3,037	37.4	35.2–39.6
Employment	Employed	7,429	40.1	38.8–41.4
	Not employed	4,646	34.0	32.4–35.6
Marital status	Married	9,806	38.3	37.0–39.6
	Not married	2,269	36.9	34.6–39.2
Region	Urban	9,322	37.6	36.5–38.8
	Rural	2,753	40.0	37.8–42.2

Weighted prevalence %, with 95% CI; n unweighted.

### Multivariate Regression Analysis

To examine the independent association between socioeconomic status and obesity, logistic regression models were estimated (Table 4). All models accounted for complex survey design and applied sampling weights. In the fully adjusted model, women had substantially lower odds of obesity than men. Compared to adults with college+ education, those with elementary education or less had significantly higher odds of obesity (OR 1.55). Income gradients were small and not statistically strong after adjusting for other SES measures. Several occupation categories showed higher odds compared to the reference group (occupation=7: unemployed/housewives/students), suggesting heterogeneity by work type and social positioning. Rural residence was not strongly associated after full adjustment.

**Table 4:** Weighted Logistic Regression Predicting Obesity (BMI ≥25), KNHANES 9^th^ Wave (2022–2024)

**Predictor**	**Model 1 OR (95% CI)**	**Model 2 OR (95% CI)**	**Model 3 OR (95% CI)**
Female (ref male)	0.52 (0.48–0.57)	0.51 (0.47–0.56)	0.52 (0.47–0.57)
Age (per year)	1.01 (1.00–1.01)	1.00 (1.00–1.00)	0.99 (0.99–1.00)
**Education (ref College+)**			
Elementary or less	—	1.60 (1.37–1.87)	1.55 (1.31–1.82)
Middle school	—	1.26 (1.07–1.50)	1.24 (1.04–1.48)
High school	—	1.06 (0.96–1.18)	1.04 (0.94–1.16)
**Income (ref Highest)**						
Lowest	—	1.03 (0.92–1.16)	1.05 (0.92–1.19)
Lower-middle	—	1.04 (0.94–1.16)	1.05 (0.93–1.19)
Upper-middle	—	1.01 (0.92–1.12)	1.01 (0.91–1.12)
**Occupation (ref occp=7 not employed)**						
Managers/professionals	—	—	1.12 (0.99–1.27)
Office workers	—	—	1.34 (1.16–1.54)
Service/sales	—	—	1.29 (1.13–1.48)
Agri/forestry/fishery	—	—	1.24 (1.00–1.55)
Crafts/operators	—	—	1.24 (1.07–1.43)
Simple labourers	—	—	1.10 (0.97–1.25)
Married (ref not married)	—	—	1.06 (0.95–1.17)
Rural (ref urban)	—	—	1.02 (0.91–1.15)

Odds ratios (OR), 95% CI.

Model 1: age + sex.

Model 2: + education + income.

Model 3 (full): + occupation + marital status + region.

Educational attainment emerged as the most consistent socioeconomic predictor of obesity. Compared with individuals with college or higher education, those with elementary education or less had significantly higher odds of obesity in both partially and fully adjusted models, while those with middle school education also showed elevated but smaller effects. These findings indicate that lower educational attainment is associated with increased obesity risk even after adjusting for income, employment, marital status, region, age, and sex. By contrast, household income showed weaker and less consistent associations with obesity. Although lower-income groups had slightly higher odds than the highest income group, differences were modest and not statistically strong in fully adjusted models, suggesting that educational attainment may be a more robust indicator of life-course socioeconomic disadvantage than current incomealone.

Employment status and occupational category demonstrat­ed heterogeneous associations with obesity. Compared with the non-employed reference group, several occupational categories, including office workers and service and sales workers, showed higher odds of obesity. These patterns suggest that work-related factors, including sedentary work environments and occupational stress, may contribute to obesity risk among employed adults. Marital status showed limited association with obesity after adjustment for other socioeconomic factors, while rural residence was not strongly associated with obesity after full adjustment. Together, these findings suggest that socioeconomic and demographic factors may play a more substantial role in obesity risk than household structure or geographic location alone.

Overall, the findings highlight that multiple dimensions of socioeconomic status contribute to obesity risk among Korean adults, with educational attainment emerging as the most consistent predictor. The results support a life-course perspective in which cumulative socioeconomic conditions shape health outcomes [36].

## Discussion

Using data from the ninth wave of KNHANES (2022–2024), this study assessed socioeconomic patterning of adult obesity in South Korea from a life-course perspective. The results indicate that obesity (BMI ≥25 kg/m^2^) remains common among Korean adults and is socially patterned in ways consistent with the life-course perspective that health is shaped by cumulative exposures and structurally patterned opportunities [37]. The analysis shows that socioeconomic indicators are not interchangeable: educational attainment is the most consistent predictor, while household income is weaker after adjustment, and occupational categories show heterogeneous associations that may reflect differences in work-related constraints and environments. From a life-course perspective, these findings are consistent with the possibility that earlier socioeconomic positioning, proxied in part by educational attainment, contributes to later socioeconomic circumstances and health-related exposures associated with obesity risk. In this view, obesity is not simply the result of individual choices but also reflects broader social and structural inequalities shaping opportunities for health.

Because the analytical sample includes adults aged 19 years and older, the findings should also be interpreted in light of life-stage heterogeneity. Early adulthood, midlife, and older adulthood are associated with different socioeconomic exposures, employment patterns, family responsibilities, and health risks. For example, educational attainment may have different implications for younger adults still transitioning into the labour market than for midlife or older adults whose occupational and income positions are more established. Similarly, obesity risk may reflect different mechanisms, including work-related sedentary behaviour in midlife and age-related health selection in older populations. These differences further highlight the importance of future linked or longitudinal data capable of examining how socioeconomic exposures and obesity risk evolve across different stages of the life-course. Future studies using linked or longitudinal data may additionally benefit from age-stratified analyses to better capture life-stage-specific socioeconomic mechanisms associated with obesity risk.

### High Overall Obesity Prevalence: Why Population-Level Framing Matters

The observed prevalence underscores obesity as a population health issue rather than a niche clinical concern. This matters for the special issue theme because life-course approaches emphasise optimising health and wellbeing across ages, not merely managing disease at a single stage. When obesity is prevalent across broad segments of the population, even modest socioeconomic gradients translate into large absolute numbers of affected individuals, reinforcing the importance of structural prevention strategies (e.g., workplace and community design, food environments, prevention-oriented primary care) rather than relying exclusively on individual behaviour change. At the same time, a life-course view implies that obesity prevention should be staged: upstream interventions targeting early-life socioeconomic conditions (education, early adult transitions into labour markets) and midstream interventions that target working-age constraints (workplace sedentary exposure, time poverty, occupational stress) are both relevant [38]. Sex differences were pronounced in the multivariable models, with women showing substantially lower odds of obesity than men under the BMI ≥25 definition. This gap likely reflects a combination of behavioural, occupational, social, and cultural factors that differ by sex in South Korea. From a life-course standpoint, these differences can be understood as the result of gendered patterns in education-to-work transitions, differential exposure to workplace environments, and differences in health behaviour norms and weight-related social pressures [39]. Crucially, this large sex difference also signals that one-size-fits-all interventions may be inefficient. Policies and programmes aiming to improve obesity-related health outcomes may need sex-sensitive design, particularly in occupational settings, if the exposure patterns and constraints differ across men and women. Future research may benefit from sex-stratified analyses to examine whether socioeconomic influences on obesity operate differently among men and women across the life-course.

#### Education as the Most Consistent SES Predictor: Why it Behaves like a Life-Course Marker

Educational attainment showed the most robust association with obesity: individuals with lower education had higher odds of obesity compared with college+ even after adjustment. This is precisely what one would expect if education operates as a foundational, early-established life-course stratifier [20, 40]. Education is typically completed earlier than many adult SES markers and tends to remain stable. It influences entry into occupational classes, shapes health literacy and the ability to interpret health information, affects income potential and job stability, and contributes to the development of social networks and norms that influence diet and physical activity. Educational attainment may also affect an individual’s ability to navigate healthcare systems and access preventive services. In life-course terms, education is not just ‘one more covariate.’ It often captures the *long shadow of early conditions* and the *structural sorting* that affects exposures for decades. The persistence of education effects after controlling for income and occupational status is consistent with the cumulative socioeconomic influences that are not fully captured by current income levels in a cross-sectional snapshot. From a research perspective, this finding suggests that educational attainment may serve as a useful anchor for approximating life-course socioeconomic positioning when longitudinal data are unavailable. From a policy perspective, the results indicate that addressing obesity inequalities may require interventions that extend beyond adult lifestyle modification and also incorporate educational equity, lifelong learning opportunities, and health literacy promotion.

#### Why Household Income Looked Weaker After Adjustment

Income gradients were modest and not strongly persistent in the fully adjusted models. This should not be interpreted as meaning material resources are irrelevant to obesity; rather, it may reflect how income functions in this dataset and context [18]: Income is more time-varying than educational attainment, and a cross-sectional measure may therefore not fully capture longer-term material conditions, particularly in the context of temporary unemployment, retirement transitions, short-term earnings fluctuations. Socioeconomic influences on health are often shaped by sustained patterns of resource access rather than income measured at a single point. Income and education are also closely interconnected. Educational attainment strongly influences employment opportunities and long-term earning potential [13, 20]. Consequently, once education is included in the model, the remaining variation in income may reflect more transient, heterogeneous, or context-specific socioeconomic circumstances. In addition, the relationship between income and obesity may additionally be more complex in advanced economies, where income differences may matter less than work constraints, sedentary exposure, and food environments that cut across income groups. Several measurement-related limitations should also be considered. Income quartiles are relative measures and may mask substantial heterogeneity within categories. Furthermore, household-level income may not accurately reflect individual access to or control over economic resources, which can vary by sex and household structure. The South Korean context may also help explain the relatively weak income gradients observed in this study. South Korea is characterised by high levels of educational attainment and intense labour market competition, conditions under which educational credentials may be more strongly linked to long-term socioeconomic opportunities than current income measured at a single point in time [9, 21]. In addition, long working hours, occupational demands, and workplace environments may influence obesity risk independently of income [39]. Together, these factors may contribute to the weaker association between current household income and obesity observed in the present analysis.

#### Occupational Patterns: What the Heterogeneity Likely Reflects

Occupational categories showed heterogeneous associations relative to the non-employed group (housewives/students, etc.). Several employed categories had higher odds of obesity, which might appear counterintuitive if employment is assumed to be uniformly protective. However, a life-course interpretation suggests more nuanced mechanisms [41]. Office-based work can increase prolonged sitting time and reduce occupational physical activity, while service/sales roles may involve irregular hours, shift work, and constrained time for exercise and meal planning. In addition, job strain and low control can influence sleep, stress-related eating behaviours, and broader metabolic dysregulation. The composition of the non-employed category should also be interpreted cautiously, as it includes heterogeneous groups such as students, homemakers, unemployed individuals, retirees, and other older adults outside the labour market. These groups may differ substantially in age, socioeconomic circumstances, health status, and obesity risk, potentially affecting comparisons across occupational categories. Future studies using more detailed labour market classifications may help disentangle these differences.

#### Marital Status: Why the Association is Small After Adjustment

Marital status showed only modest association with obesity after controlling for socioeconomic variables. This may reflect competing mechanisms. Marriage can increase household stability and regular meal patterns, but can also reduce leisure-time physical activity for some individuals, and interact with parenthood and caregiving responsibilities. Once education, occupation, and age are controlled, marital status may offer limited independent explanatory power. From a life-course perspective, marital status may act more as a transition marker of social and family circumstances across different stages of adulthood than as a primary structural determinant of obesity risk. Similarly, regional differences were not strongly associated with obesity after full adjustment. Although rural residents showed slightly higher obesity prevalence, these differences appear to be largely explained by socioeconomic composition and occupational structure rather than geography alone. Alternatively, it may indicate that macro-level drivers (food environments, sedentary lifestyles, widespread access to calorie-dense foods) operate broadly across regions in South Korea. Together, these results suggest that social and environmental contexts remain relevant, but their effects may be mediated through broader socioeconomic processes. Future linked data research incorporating neighbourhood characteristics, residential histories and household composition could provide a more nuanced understanding of these contextual influences across the life-course.

### WHO Life-Course Framework and Linked Data Implications

A WHO-aligned life-course approach emphasises maintaining and enhancing functional ability and wellbeing across all ages through person-centred and multisectoral strategies [38]. This perspective is closely aligned with the WHO Framework for Implementing a Life-Course Approach in Practice, which emphasises integrated, multisectoral, and prevention-oriented approaches to health across the lifespan. The framework highlights the importance of addressing social and structural determinants of health throughout different stages of life while strengthening data systems to support longitudinal research and evidence-based life-course policy [42]. The findings of this study align closely with this framework and demonstrate how nationally representative health survey data can inform life-course health analysis even in the absence of fully linked longitudinal datasets.

This study supports a life-course approach in several important ways. First, the persistent association between educational attainment and obesity highlights the importance of life-course interpretation [14, 37]. Educational attainment, typically established early in adulthood and remaining relatively stable thereafter, shapes long-term socioeconomic positioning, employment opportunities, and health behaviours. The strong and consistent relationship observed between lower education and higher obesity risk is consistent with the possibility that earlier social positioning contributes to later socioeconomic and health-related exposures associated with obesity risk. These findings are broadly consistent with life-course perspectives emphasising pathway mechanisms through which earlier socioeconomic conditions shape later opportunities, constraints, and health-related exposures associated with obesity risk. Second, the heterogeneity observed across occupational categories underscores the importance of multisectoral leverage points for health policy. Differences in obesity risk across occupational groups suggest that workplace environments, labour conditions, and time constraints play meaningful roles in shaping health behaviours and metabolic outcomes. These findings point to the workplace and labour policy domain as key intervention spaces extending beyond traditional health services. From a life-course perspective, occupational environments represent sustained exposures that may influence adult health outcomes over time. Third, the results support the need for prevention across life stages rather than focusing solely on late-stage clinical management of obesity. The persistence of socioeconomic gradients is consistent with the possibility that structural determinants operating across education, employment, and social environments shape obesity risk long before clinical intervention occurs. Upstream and midstream interventions targeting educational inequalities, workplace conditions, and broader social determinants of health are therefore essential components of a comprehensive life-course strategy.

**Table 5:** Empirical Findings, Life–Course Interpretation, and Linked Data Implications

**Empirical finding**	**Life-course interpretation**	**Linked data potential**
Education strongly associated with obesity	Early-established socioeconomic positioning shapes long-term health outcomes	Link with education histories and early-life SES indicators
Income effects modest after adjustment	Cross-sectional income may not capture cumulative material conditions	Longitudinal income and employment records
Occupational heterogeneity	Workplace exposures and constraints influence adult health outcomes	Employment histories, working hours, job strain indicators
Modest marital and regional effects	Household and geographic context interact with structural SES factors	Household composition and neighbourhood environment data
High overall obesity prevalence	Population-level exposure to obesogenic environments across life stages	Longitudinal health and service utilisation records

Beyond its substantive findings, this study highlights the relevance of linked data for advancing life-course research. Although KNHANES is cross-sectional, it can support analyses within a life-course framework through relatively stable socioeconomic indicators such as education with adult structural measures including occupation and income. Expanding linked data infrastructures would further strengthen life-course research.

KNHANES can be positioned as a central platform within an emerging linked data ecosystem in several ways. First, it can function as a hub dataset for enrichment [43]. KNHANES provides high-quality measured health outcomes and detailed socioeconomic information. Linking it with administrative and sectoral datasets would enable more comprehensive analyses of weight patterns, chronic disease onset, healthcare utilisation, employment histories, and environmental exposures across the life-course. Second, KNHANES can serve as a governance and methodological testbed for developing privacy-preserving linkage models that balance research utility with confidentiality. At the same time, several governance and methodological challenges continue to limit broader linked data development in South Korea. Administrative and health-related datasets are often managed by separate institutions with differing access procedures, governance structures, and privacy regulations, which may complicate inter-agency linkage and data harmonisation. In addition, concerns regarding confidentiality, secure data sharing, and long-term governance frameworks may limit broader research accessibility to integrated datasets. These challenges highlight the importance of developing secure and transparent linkage infrastructures capable of balancing data protection with research utility in future life-course research initiatives. Third, the Korean experience offers a transferable model for countries without mature linked data systems, demonstrating how life-course analyses can begin with existing national surveys while progressively expanding linkage capacity. In this sense, KNHANES illustrates how routinely collected survey data can support life-course research and inform policy even where fully integrated longitudinal systems remain under development. To synthesise these implications, Table 5 summarises how the empirical findings of this study relate to life-course interpretation and to future data linkage opportunities.

## Conclusion

This study examined socioeconomic positioning and adult obesity using nationally representative data from the ninth wave of the Korea National Health and Nutrition Examination Survey (KNHANES, 2022–2024). By applying a life-course perspective to cross-sectional population health data, the study provides evidence consistent with cumulative socioeconomic influences on obesity risk among adults in South Korea using proxy indicators of socioeconomic positioning. The study also highlights the analytical value of national health surveys for life-course research. Several strengths should be noted. First, the study uses recent nationally representative data with comprehensive coverage of the adult population, enhancing the generalisability of findings. The use of measured anthropometric data ensures accurate classification of obesity and strengthens the reliability of results. Second, a multidimensional approach to socioeconomic status was adopted by examining education, income, occupation, and social context simultaneously. This enables a more comprehensive assessment of how cumulative socioeconomic conditions influence obesity risk. Third, situating the analysis within a life-course framework extends beyond conventional cross-sectional approaches and highlights the importance of long-term socioeconomic conditions and structural influences in shaping adult health outcomes. An additional strength lies in demonstrating how routinely collected national health survey data can be leveraged to examine socioeconomic patterns in population health. In settings where fully linked longitudinal datasets remain limited, national surveys such as KNHANES provide valuable resources for examining structural determinants of health and identifying inequalities across populations. This study therefore illustrates the potential of existing data infrastructures to support life-course analysis and inform policy discussions.

Several limitations should also be acknowledged. First, the cross-sectional design of KNHANES limits the ability to establish causal relationships or directly observe changes in socioeconomic conditions and health outcomes over time. Although the life-course perspective offers a useful interpretive framework, longitudinal data would be required to fully capture long-term socioeconomic transitions and exposures across different stages of life. In addition, some relevant life-course factors, including childhood socioeconomic conditions and early-life health exposures, are not directly measured and therefore must be approximated through adult indicators such as educational attainment. The inclusion of older adults requires caution in interpreting BMI-based obesity. In older populations, associations between BMI and health outcomes may be affected by age-related changes in body composition, survival selection, and the so-called obesity paradox, whereby higher BMI may appear less harmful or even protective for some outcomes. Health survey participation among older adults may additionally be affected by healthy participant bias, as frailer individuals or those with more severe illness may be less likely to participate. Furthermore, the measurement of income and occupation at a single time point may not fully reflect long-term socioeconomic experiences, while the non-employed category includes heterogeneous groups such as students, homemakers, and retirees, potentially masking differences in socioeconomic conditions. Despite these limitations, the dataset provides valuable insight into the socioeconomic patterning of obesity within the adult population of South Korea.

This study contributes to the literature on life-course health inequalities by demonstrating the value of integrating life-course perspectives with nationally representative survey data. It provides empirical evidence of persistent socioeconomic inequalities in obesity in South Korea and highlights educational attainment as a key determinant of adult obesity risk. Methodologically, it shows how cross-sectional survey data can support life-course analysis using proxy indicators of socioeconomic positioning measured while also informing future linked data development. Future research should build on these findings by incorporating longitudinal and linked data sources that enable more detailed examination of health patterns and socioeconomic influences. Linking national health survey data with administrative and sectoral records, including National Health Insurance Service (NHIS) data, healthcare utilisation records, mortality data, employment histories, educational records, and neighbourhood or built-environment data, would substantially strengthen the evidence base for life-course research and policy development. Such linkages would enable more direct examination of socioeconomic exposures, cumulative disadvantage, healthcare utilisation patterns, and health outcomes across different stages of life. The present findings may also help inform the design of future linked data studies by identifying key socioeconomic indicators and population groups relevant to obesity-related health inequalities in South Korea. Overall, the findings underscore the importance of integrated data systems and multisectoral approaches to improving population health across the life-course.

## Data Availability

The data that support the findings of this study are publicly available from the Korea National Health and Nutrition Examination Survey (KNHANES) and can be accessed through the official Korea Disease Control and Prevention Agency (KDCA) website (https://knhanes.kdca.go.kr) upon reasonable request and registration.
